# Detection and Dispersion Analysis of Water Globules in Oil Samples Using Artificial Intelligence Algorithms

**DOI:** 10.3390/biomimetics8030309

**Published:** 2023-07-13

**Authors:** Alexey N. Beskopylny, Anton Chepurnenko, Besarion Meskhi, Sergey A. Stel’makh, Evgenii M. Shcherban’, Irina Razveeva, Alexey Kozhakin, Kirill Zavolokin, Andrei A. Krasnov

**Affiliations:** 1 Department of Transport Systems, Faculty of Roads and Transport Systems, Don State Technical University, 344003 Rostov-on-Don, Russia; 2 Strength of Materials Department, Faculty of Civil and Industrial Engineering, Don State Technical University, 344003 Rostov-on-Don, Russia; 3 Department of Life Safety and Environmental Protection, Faculty of Life Safety and Environmental Engineering, Don State Technical University, 344003 Rostov-on-Don, Russia; spu-02@donstu.ru; 4 Department of Unique Buildings and Constructions Engineering, Don State Technical University, 344003 Rostov-on-Don, Russia; sergej.stelmax@mail.ru (S.A.S.); razveevai@mail.ru (I.R.); alexeykozhakin@gmail.com (A.K.); 5 Department of Engineering Geology, Bases, and Foundations, Don State Technical University, 344003 Rostov-on-Don, Russia; au-geen@mail.ru; 6 OOO VDK, SKOLKOVO, Bolshoi Boulevard, 42, 121205 Moscow, Russia; kirillzavolokin@yandex.ru (K.Z.); akrasnov@vdktech.ru (A.A.K.)

**Keywords:** artificial intelligence, convolutional neural network, computer vision, detection, oil, oil dehydration, water globules

## Abstract

Fluid particle detection technology is of great importance in the oil and gas industry for improving oil-refining techniques and in evaluating the quality of refining equipment. The article discusses the process of creating a computer vision algorithm that allows the user to detect water globules in oil samples and analyze their sizes. The process of developing an algorithm based on the convolutional neural network (CNN) YOLOv4 is presented. For this study, our own empirical base was proposed, which comprised microphotographs of samples of raw materials and water–oil emulsions taken at various points and in different operating modes of an oil refinery. The number of images for training the neural network algorithm was increased by applying the authors’ augmentation algorithm. The developed program makes it possible to detect particles in a fluid medium with the level of accuracy required by a researcher, which can be controlled at the stage of training the CNN. Based on the results of processing the output data from the algorithm, a dispersion analysis of localized water globules was carried out, supplemented with a frequency diagram describing the ratio of the size and number of particles found. The evaluation of the quality of the results of the work of the intelligent algorithm in comparison with the manual method on the verification microphotographs and the comparison of two empirical distributions allow us to conclude that the model based on the CNN can be verified and accepted for use in the search for particles in a fluid medium. The accuracy of the model was *AP@*50 = 89% and *AP@*75 = 78%.

## 1. Introduction

Global and Russian experiences with oil refineries show that one of the most significant and complex technological processes in the processing of an oil emulsion is the separation of the ballast (water and substances dissolved in it) from the oil. Insufficient efficiency in the process of dehydrating oil supplied for transportation and processing can lead to the corrosion of equipment and reductions in the service lives of process units and, as a result, worsens the quality-determining characteristics of the resulting oil products [1]. In this regard, the development of methods and tools for automating and digitalizing the oil dehydration process is an urgent task for the entire oil industry. The works [2,3,4] consider the microwave method, and in [5], the ultrashort wave method, which allows for the measurement of both high and low water contents in crude oil in an oil pipeline, is considered. The use of spectroscopic detection technology to measure the water content in crude oil was shown by researchers from China [6,7]. With the help of POF sensors, the water content in a water–oil emulsion (WOE) is considered in [8]. In addition, the water content of crude oil can be measured using a cavity resonator [9].

The near-infrared range, along with the partial least squares method, has been used to estimate the size distribution of water droplets in crude oil emulsions in a pressurized pipeline. The authors noted its high performance in that the models achieved for the pressurized system presented good predictions of the properties of the emulsion and its water content. The provided instrument for the online detection of water droplets via size and water content in crude oil emulsions in a pressurized pipeline has shown potential for real-world applications [10]. In addition, many studies have considered the detection of water droplets in solutions [11] and the separation of oil and water [12] using the electrochemical method [11], 2D materials [13], and electrospinning membranes [14]. The sizes and shapes of the particles affect the quality of the product. Real-time control of such characteristics in a multiphase system is difficult due to the low measurement accuracy of the existing instruments. However, the development of photo-optical methods now allows for accurate real-time measurements of the size, shape, and concentration of particles separated from each other in three-phase systems [15].

Recently, the concept of a “smart field” has been actively spreading, within the framework of which innovative technologies of intellectualization in the area of oil and gas production are being developed. An increasing number of “smart” algorithms based on computer vision are being introduced as systems for obtaining, processing, and monitoring data at all stages of the oil refining complex, including the dehydration of an oil emulsion [16,17]. The integration of methods and tools of artificial intelligence makes it possible to automate routine, labor-intensive, or computationally complex processes, as well as to increase their accuracy and reduce their speed of execution and the influence of the human factor [18]. Artificial intelligence methods are already being applied to predict the molecular weight of heavy oil. It is noted that an artificial neural network can predict oil characteristics with greater accuracy than regression models [19]. The gamma attenuation method, wavelet characteristics, and neural networks make it possible to determine and predict the types and different volume percentages of oil products inside a scaled pipe of different thicknesses. This will optimize the system and increase the productivity of the oil industry [20,21,22]. The issues of detecting oil on the surface of water [23] and underwater [24], the autonomous monitoring of oil slicks [25,26], and the separation of oil from water with the removal of heavy metals [27] have also received a significant amount of attention in modern research. In addition, there are studies on the possibility of predicting the distribution of water saturation in reservoirs [28], as well as predicting and monitoring various properties of oil products using artificial intelligence [29,30,31]. In [32], an algorithm for automatic counting and the measurement of particles in multiphase systems was implemented in MATLAB version 7.9 (R2009b). The program achieved a 95% match rate, with an error rate of less than 1% and a detection rate of 250 particles per minute depending on the system. In [33], the process of quantitatively analyzing polymer particles suspended in aqueous media was considered. The calculation was carried out using a special computer program developed in the language Visual Basic (VB) which calculates the area of the particles from the total area.

This review analysis presents the increasing relevance and popularity of the introduction of intelligent technologies which have already proven themselves in various industries [34,35,36,37] into the oil industry; for example, the use of e-management tools for the intellectualization of the production [38], analysis, and prediction of crude oil prices based on machine learning methods [39,40,41] and the use of intelligent algorithms to evaluate the efficiency of equipment and processes in production [42,43,44] and in the assessment of various characteristics of oil products [45,46]. There are also studies describing the process of water segmentation in images using a convolutional neural network (CNN) [47]. A convolutional neural network is effectively trained to detect particles, providing high-quality particle recognition for subsequent size and shape analyses under various experimental conditions [48]. However, as a result of an analysis of the literature, a shortage of works on the combined use of microscopy and machine learning methods to detect water droplets and their sizes in crude oil was revealed. The scientific novelty of this research lies in the following:

− The application of an integrated method for detecting water globules in crude oil based on a microscopic method and computer vision, which allows for the effective automation and digitalization of the process of oil dehydration;
− The creation of an empirical base of WOE microphotographs at characteristic points of the technological scheme, including the use of in situ microscopy technologies;
− The expansion of the training sample using the authors’ augmentation algorithm;
− The development of a computer vision algorithm to detect water globules in crude oil;
− The optimization of the characteristics of the intellectual model based on the CNN YOLOv4;
− An analysis of variance with respect to the size of detected particles;
− A comparison of two empirical distributions obtained via manual detection method and the neural network method by calculating the Kolmogorov–Smirnov criterion.

In the present work, with the use of elements of computer vision, the detection of water globules is implemented, and a dispersion analysis of their sizes is also carried out. The main goal was to implement, debug, optimize, and test a detector based on the YOLOv4 convolutional neural network. The study completed the following tasks:

− Collecting an empirical base of WOE microphotographs;
− Substantiating and describing the chosen convolutional neural network, YOLOv4;
− Expanding of the data set using the augmentation algorithm;
− Implementing, debugging, and optimizing the developed detector;
− Testing the detector and determining quality metrics for the detection of water globules in the WOE;
− Identifying the nature of the WOE at key points of the technological scheme of the electric desalination plant (ELDP) by conducting a dispersion analysis of the size of the detected particles;
− Comparing two empirical distributions using the Kolmogorov–Smirnov criterion.

The theoretical significance of the study lies in the expansion of ideas about the possibilities of using computer vision algorithms in the intellectualization of the oil industry. The practical significance of the work lies in the development of an applied and scalable algorithm within the concept of a “smart field”.

## 2. Materials and Methods

### 2.1. Materials and Stages of the Study

Crude oil is a stable inverted polydisperse emulsion in which formation water is distributed in the form of globules. The volume concentration of water in oil is 0.1–2%. The stability of emulsions, in addition to the physical and chemical properties of raw materials, is also determined by the size of the water globules and the lifetime of the emulsion.

The deep dehydration of oil reduces transportation costs and the amount of wastewater discharged by oil refineries. Therefore, the control of the dispersion band and the determination of the average size of the globules is the most important step in achieving petroleum products of the desired quality.

The preparation of an optimal WOE becomes critical to guaranteeing the protection of equipment from corrosion and ensuring the stability of the main technology since a forced increase in the consumption of alkali to neutralize corrosive agents will inevitably lead to an increase in the sodium content in the feedstock after ELDP.

**Figure 1:**
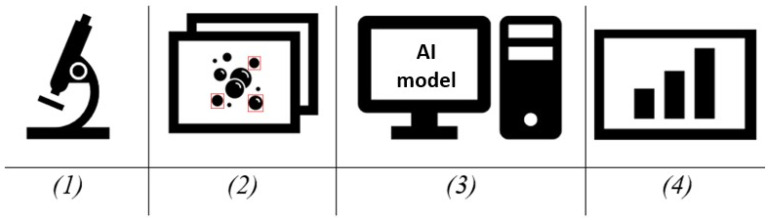
The sequence of the stages of the study: (1) the preparation of the empirical base for the study; (2) markup and data augmentation; (3) the implementation, optimization, and testing of an intelligent algorithm; (4) the interpretation of the results.

This study proposes a solution for the detection of water globules in an oil emulsion and an analysis of their variance using an artificial intelligence method, specifically, a computer vision method based on the CNN YOLOv4. The sequence of the research stages is shown in Figure 1.

### 2.2. Preparing Data for an Intelligent Algorithm

**Figure 2:**
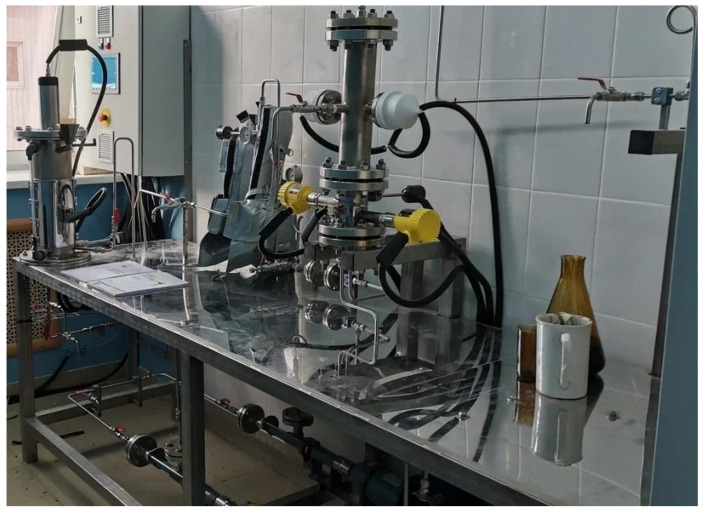
Conducting laboratory tests.

The data source for this study comprises the results of a study by specialists from the research and production company OOO VDK (a resident of the Skolkovo Innovation Center), which were obtained during work on the WOE static mixer S-Mix-DS preparation complex [49,50], as well as during laboratory tests reproducing real operating conditions for elements of the mixer (Figure 2). Previously, OOO VDK used a manual method for the analysis of water–oil emulsions.

As an empirical base, 20 microphotographs of a WOE at characteristic points of the technological scheme, including those using in situ microscopy technologies, were used. The sampling was carried out using a hardware–software complex, including a Levenhuk D320L PLUS microscope (Levenhuk, St. Petersburg, Russia) with a resolution of 2048 × 1536 and a computer. When photographing, the presence of a significant light gradient should be avoided, since the latter significantly complicates further computer processing of the image and interpretation of the results.

**Figure 3:**
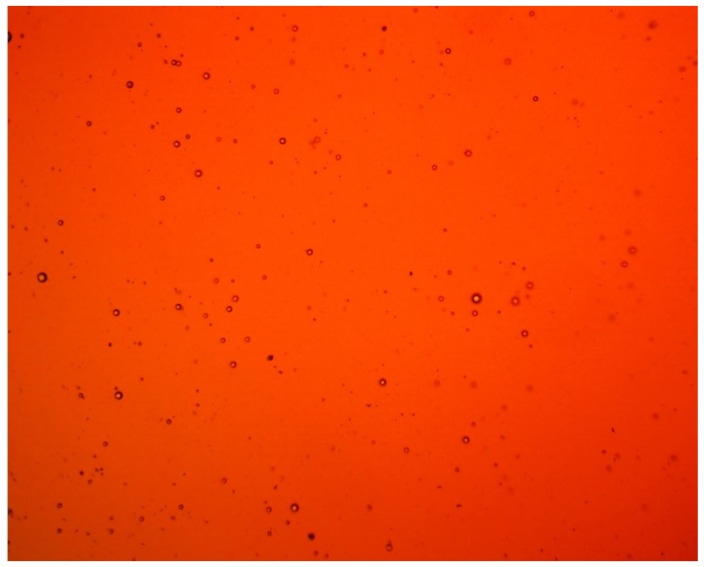
Micrograph of crude oil.

Crude oil contains formation water in the form of a finely dispersed emulsion with an average microdroplet size of 7–8 µm. At the same time, a high content of globules 2–4 µm in size is observed (Figure 3).

### 2.3. Augmentation and Labeling of Crude Oil Micrographs 

To represent the images as input data for the input of the neural network algorithm, it is necessary to markup water globules using bounding boxes. The markup was completed using the application Image Labeler, which provides a way to interactively create different shapes to label areas of interest.

Deep learning requires a large training set to obtain good model generalization results and reduce the effect of overfitting. If it is difficult to increase the volume of real data, researchers apply various methods of data inflation based on the available data [51]. In this study, the increase in the size of the original set was artificially carried out using the authors’ augmentation algorithm in which for each image, the coordinates of the bounding box are automatically recalculated [52].

The number of annotated images in the training sample was increased from 20 to 500. The possible modifications that carried out on the images before training the model are as follows.

**Figure 4:**
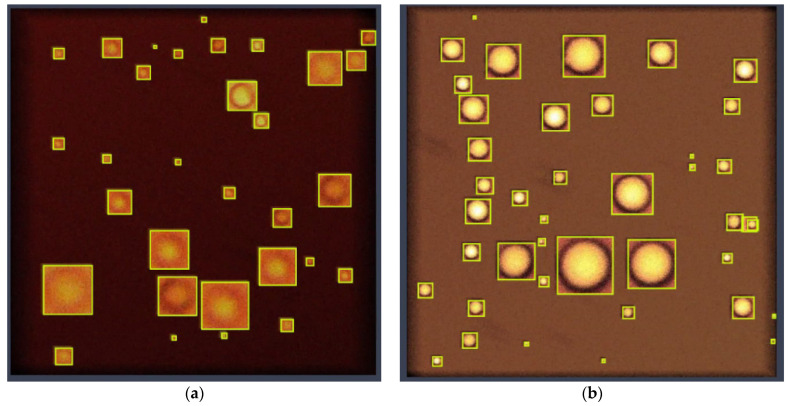
Operations with color during image augmentation: (**a**) change in brightness; (**b**) change in saturation.

1. Operations with color: changing the contrast, brightness, and saturation (Figure 4).
2. Geometric operations: image rotation/reflection, random image shifts along the Ox and Oy axes, and image rotation by 90°, 180°, and 270° [53,54].

Training on augmented data, which is a more complete set of possible variants of input images, will make it possible to achieve model stability against sudden noises in case they appear during photo capture.

### 2.4. The Implementation of an Intelligent Algorithm

**Figure 5:**
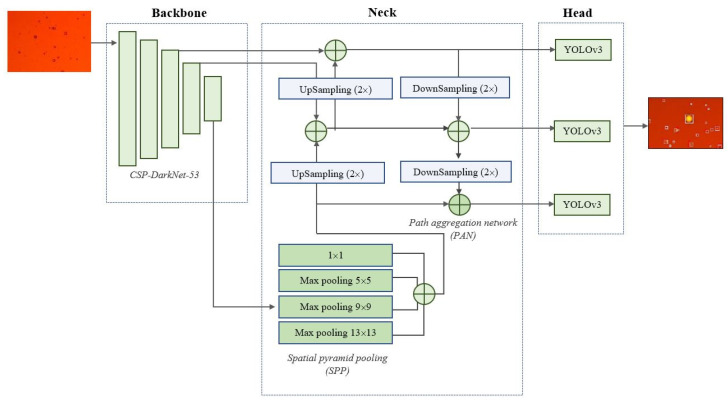
YOLOv4 CNN architecture.

In this study, a detector from the YOLO family, which belongs to the family of so-called state-of-the-art (SotA) advanced technologies, was used to detect water globules in oil. The single-stage YOLOv4 detector was chosen due to the fact that the architectural implementation of the algorithm implies the efficient use of computing resources, while the quality of the recognition of small objects in the object image is improved (Figure 5) [55].

The parameters that were changed during the study included the size of the training, validation, and test sets and the batch size, which were selected based on the limitations of the computing resources in terms of memory; the number of epochs, determined upon reaching the required accuracy and the time of access to computing resources; and the learning rate and optimizer at which the model showed the best quality.

The YOLOv4 architecture uses CSPDarknet53 as its backbone. In the neck block, feature aggregation occurs: “SPP (Spatial al pyramid pooling) is a method of acquiring both fine and coarse information by simultaneously pooling on multiple kernel sizes (1, 5, 9, 13)”. The main role of PANet is to improve the instance segmentation process by storing spatial information, which in turn aids with correct pixel localization for mask prediction. “The head processes the aggregated features and predicts the bounding boxes, objectness scores, and classification scores. The YOLO v4 network uses one-stage object detectors, such as YOLOv3, as detection heads” [56].

An important step in training the YOLOv4 CNN is the selection of its parameters (Table 1).

The data set was divided into training, validation, and test sets at the ratio 70/15/15. The “Batch Size” parameter determines the number of examples used in one epoch to train the neural network. The learning rate is one of the most important hyperparameters and allows the user to control the amount of weight correction at each iteration. The stochastic gradient descent (SGD) was used as an optimizer.

**Figure 6:**
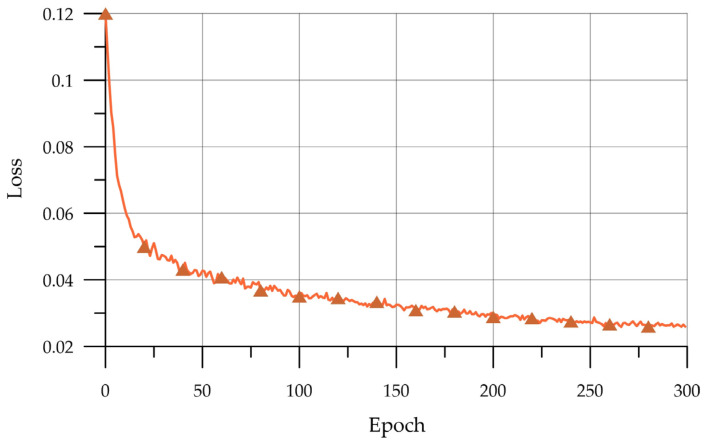
CNN training.

The intellectual model was trained on a high-performance OOO VDK cluster based on a two-processor server with an installed Intel (Santa Clara, CA, USA) Xeon E5-2683 v3 processor. The training time was 52 min. The model training graph is shown in Figure 6, in which the y-axis shows the error values between the predicted and actual bounding box, and the abscissa shows the epochs.

From the graph of the value of the error function during the training of the CNN, it follows that up to about 250 epochs, the error steadily decreases, but then it changes slightly, which indicates sufficient training.

## 3. Results and Discussion

**Figure 7:**
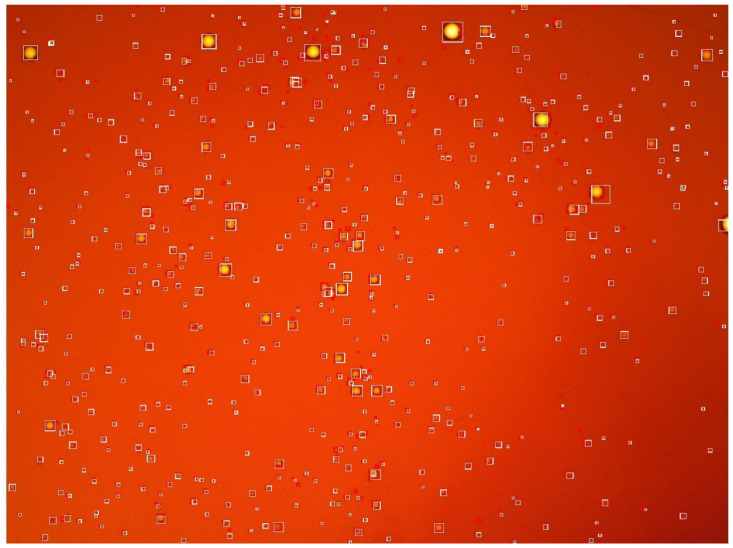
Result of the detection of water globules in oil.

The developed intellectual algorithm based on the CNN YOLOv4 makes it possible to detect particles in a fluid medium at the level of accuracy required by the researcher, which can be controlled at the stage of training the CNN. Figure 7 shows the result of the work of the intelligent algorithm, which is an image with localized globules in the bounding boxes.

When training a CNN to identify objects of a certain class, it is necessary that the intelligent model finds these objects regardless of their location and size in the image. Figure 7 shows that the model finds globules over the entire area of the image, both in places with a large accumulation of particles of different sizes and when they are located far from each other.

To evaluate the detection quality, the following metrics are usually used: precision (*P*), recall (*R*), and average precision (*AP*).

Precision and recall can be calculated using the formulas:

(1) $$\mathrm{Precision}(P)=\frac{TP}{TP+FP}$$

(2) $$\mathrm{Recall}(R)=\frac{TP}{TP+FN}$$

where “true positive (*TP*) is the correct detection made by the model; false positive (*FP*) is incorrect detection made by the detector; false negative (*FN*) is a true result missed (not detected) by the detector” [53,54].

*AP@α* is the area under the precision–recall curve (AUC–PR) estimated at the threshold α IoU. The intersection over union (IoU) is used to evaluate the object detection performance by comparing the ground truth bounding box to the predicted bounding box. IoU values range from 0 to 1, where 0 indicates no overlap and 1 indicates perfect overlap. The IoU threshold decides whether the prediction is a true positive (*TP*), false positive (*FP*), or false negative (*FN*). The formula for the calculation is as follows:

(3) $$AP@\alpha =\int_{0}^{1}P\left(R\right)dr$$

“*AP@*50 and *AP@*75 values were calculated showing *AP* values calculated at IoU = 0.50 and IoU = 0.75 respectively” [53,54]; in this study, *AP@*50 = 89% and *AP@*75 = 78%. If the level of accuracy obtained using the indicated metrics is lower than the level of accuracy declared by the researcher (in practice, from 85%), then it is necessary to increase the number of images in the training set and/or add additional effects to the augmentation process and retrain the CNN.

Precision, recall, and average precision (*AP*) are key indicators for evaluating the effectiveness of algorithms for object detection. With a clear understanding of these metrics, one can evaluate how well the trained model solves the detection problem (Table 2).

**Figure 8:**
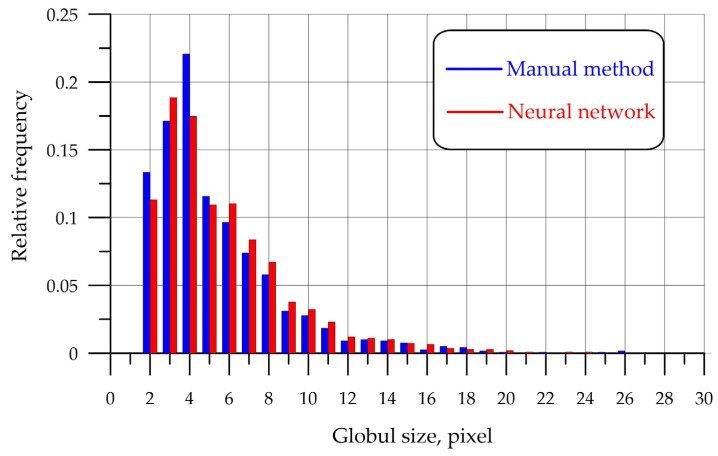
Histogram of an analysis of variance with the manual method and with the neural network method: **■**—manual method; **■**—neural network method.

Figure 8 shows a histogram comparing detection between the use of the manual detection method by a technologist and the intelligent detection method.

The number of globules detected via the manual method was 1192, and when using the neural network detection, it was 1088. The sizes of the globules are indicated along the Ox axis in pixels. When switching to standard diameter units, the following formula is used:

(4) D=p×0.629

where *D* is the globule diameter in µm, and *p* is the diameter of the globule in pixels (px).

According to Figure 8, in the presented WOE sample, the highest concentration of globules with diameters ranging from 2 to 5 μm is observed, both via the manual detection method and the neural network, which corresponds to the actual consistency of crude oil. As the sizes of the globules increase, their number decreases with the use of both methods of detection.

To compare two empirical distributions obtained via the manual method of detecting water globules in oil and the developed neural network algorithm, the Kolmogorov–Smirnov criterion was applied. This criterion allows for the identification of the point at which the sum of the accumulated discrepancies between the two distributions is the largest and for an assessment of the reliability of this discrepancy.

**Figure 9:**
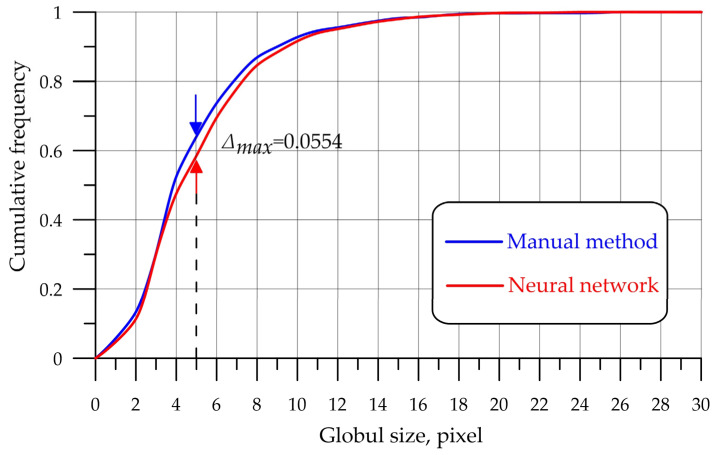
Empirical probability distribution functions for ■ manual processing and ■ CNN.

A comparison of two empirical probability distribution functions, the manual method of processing results and processing with the help of a CNN, is shown in Figure 9.

We put forward the following hypotheses:

**Hypothesis** **0** **(H0).** *Empirical distributions for the manual detection method and for the neural network method do not differ, that is, the differences between the two distributions are not significant*.

**Hypothesis** **1** **(H1).** *The empirical distributions for the manual detection method and for the neural network method are different, that is, the differences between the two distributions are significant*.

The algorithm for working with the criterion:

1. Calculate the accumulated empirical relative frequencies *N_1emp_* for the first distribution.
2. Calculate the accumulated empirical relative frequencies *N_2emp_* for the second distribution.
3. Calculate the empirical value of the criterion (5):
  (5) $$\lambda_{emp}=\max\left|N_{1emp}-N_{2emp}\right|\times \sqrt{\frac{n_{1}\times n_{2}}{n_{1}+n_{2}}}$$
  where *n*_1_ and *n*_2_ are sample sizes.
4. Define the critical points:
  (6) $$\lambda_{cr}=\left\{\begin{array}{c} 1.36,\;at\;\gamma =0.95\\ 1.63,\;at\;\gamma =0.99 \end{array}\right.$$
  where *γ* is the confidence level.
5. The conclusion is that $\lambda_{emp}\geq 1.36$; the differences between the distributions are significant.

The maximum discrepancy between the accumulated observed and theoretical frequencies is determined as follows:

(7) $$\lambda_{emp}=0.055\times \sqrt{\frac{1092\times 1088}{1092+1088}}=1.32$$

The level of statistical significance of the obtained value is *p* = 0.06.

**Figure 10:**
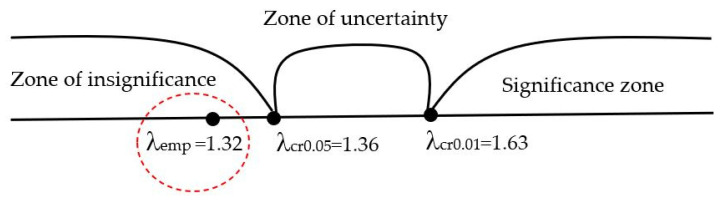
Significance axis. The colored circle highlights the criterion value of the zone of insignificance 1.32 < 1.36.

For clarity, an axis of significance was built (Figure 10).

The axis shows critical values corresponding to the accepted levels of significance: $\lambda_{cr0.05}=1.36$ and $\lambda_{cr0.01}=1.63$. To the right of the critical value $\lambda_{cr0.01}$, the “significance zone” extends. It includes empirical values exceeding $\lambda_{cr0.01}$, which are therefore unconditionally significant. To the left of the critical value $\lambda_{cr0.05}$, the “zone of insignificance” extends. It includes empirical values that are below $\lambda_{cr0.05}$, and are therefore certainly insignificant. Thus, $\lambda_{emp}$ falls into the area of acceptance of the hypothesis H_0_, which means that the differences between the two distributions are not significant. Therefore, the empirical distributions for the manual detection method and for the neural network method do not differ.

An evaluation of the quality of the results of the work of the intelligent algorithm in comparison with the manual method allows us to conclude that the model based on a CNN can be verified and accepted for use in the search for particles in a fluid medium. The developed model is suitable for various types of water–oil emulsions if the liquid has similar features at the visual structure level. If there are significant differences, then the result is not known in advance and requires additional research.

Other methodologies that can achieve the goal of the current work include the manual method, semi-automatic methods that require human participation, classical computer vision methods, and laboratory sedimentation analyses.

Comparing the developed algorithm for detecting water globules in a WOE with methods based on the use of optical micro-resonator, fiber optic, microwave, and ultrasonic sensors, it can be noted that the equipment is sensitive to the physico-chemical properties of the sample (for example, to temperature) and also requires effort when used in the field, while the proposed intelligent model depends only on the quality of the micrographs proposed for analysis. The software proposed in [32,33] is ensured to be very reliable and able to compete with the human eye in complex images. At the same time, the computational speed is quite low. Thus, in [32], the time is almost proportional to the number of processed pixels and depends on the initial characteristics of the image, while the method proposed in this study provides a result in 3–5 min. It should also be noted that in [33], there is a complexity of software scaling due to its implementation in the VB language, while the implemented intelligent model was developed in the modern, high-level language Python. The use of a CNN in computer vision problems is the most popular choice of researchers, and the YOLOv4 architecture is optimal for use in terms of its *AP* (accuracy)/*FPS* (speed) at the time of the start of this study, which has been confirmed by other authors [48].

## 4. Conclusions

The article describes the process of creating a computer vision algorithm that allows a user to detect water globules in oil samples and analyze their sizes. The algorithm was developed on the basis of the YOLOv4 convolutional neural network. For this study, the use of the authors’ own empirical base was proposed, which comprised microphotographs of samples of raw materials and water–oil emulsions taken at various points and in different operating modes of an oil refinery. The increase in the number of images (data) by creating (generating) new images for training by applying transformations to the original set of images from the training set was carried out using the authors’ algorithm. To expand the adaptive properties of the algorithm and its stable operation under various laboratory and field conditions, the authors’ algorithm was applied, thanks to which the training set was supplemented with images containing visual effects, noise, and distortion.

The following conclusions can be made on the basis of the results obtained.

(1) The developed intellectual algorithm based on the CNN YOLOv4 makes it possible to detect particles in a fluid medium with the level of accuracy required by the researcher, which can be controlled at the stage of training the convolutional neural network.

The required level of accuracy in these tasks was set to 85% by VDK technologists.

(2) An evaluation of the quality of the results of the work of the intelligent algorithm in comparison with the manual method on the verification microphotographs and comparison of two empirical distributions allows us to conclude that the model based on the SNA can be verified and accepted for use in the search for particles in a fluid medium since the empirical distributions for the manual detection method and for the neural network method do not differ.
(3) The manual marking of one microphoto by a technologist o can take up to 8 h, while the computer vision algorithm developed in this study allows for the localization of water globules in oil with an accuracy of *AP@*50 = 89% and *AP@*75 = 78% in 3–5 min.

A comparison with the manual method favors the developed intellectual model. In studies of this kind, comparison with the manual method of particle detection is the most characteristic (classical and correct). Comparisons with other modern methods are not possible as they are often commercial and require a specialist, as well as additional research.

(4) The YOLOv4 architecture allows for the problem of object detection using low-performance devices to be solved while processing incoming data in real time at high speed. Thus, the developed intelligent model can be used to solve computer vision problems directly on a smartphone or when using an autonomous robot. In addition, the developed model is suitable for various types of water–oil liquids if the liquid has similar features at the visual structure level. If there are significant differences, then the result is not known in advance and requires additional research.

The prospects of research development are to increase the training data set under laboratory and field conditions, apply additional visual effects when expanding the training dataset, and test the algorithm online using in-situ microscopy technologies.

## 5. Patents

Beskopylny, A.N.; Mailyan, L.R.; Stel’makh, S.A.; Shcherban’, E.M.; Razveeva, I.F.; Beskopylny, N.A.; Dotsenko, N.A.; El’shaeva, D.M. The program for determining the me-chanical properties of highly functional lightweight fiber-reinforced concrete based on ar-tificial intelligence methods. Russian Federation Computer program 2022668999, 14 October 2022. Available online: https://www.fips.ru/registers-doc-view/fips_servlet?DB=EVM&DocNumber=2022668999&TypeFile=html (accessed on 11 May 2023)

Beskopylny, A.N.; Stel’makh, S.A.; Shcherban’, E.M.; Razveeva, I.F.; Kozhakin, A.N.; Beskopylny, N.A.; Onore, G.S. Image Augmentation Program. Russian Federation Com-puter program 2022685192, 21 December 2022. Available online: https://www.fips.ru/registers-doc-view/fips_servlet?DB=EVM&DocNumber=2022685192&TypeFile=html (accessed on 11 May 2023)

## Figures and Tables

**Table 1:** YOLOv4 training parameters.

№	Parameter	Value
1	Number of shots in the training set	350
2	Number of shots in the validation set	75
3	Number of shots in the test set	75
4	Batch size	10
5	Number of epochs	300
6	learning rate	0.01
7	Solver	SGD

**Table 2:** Model quality estimates.

Num	Parameter	IoU = 0.50	IoU = 0.75
1	Precision	89%	74%
2	Recall	73%	64%
3	Average precision	89%	78%

## Data Availability

The study did not report any data.
